# *In-vitro* Thermal Maps to Characterize Human Dental Enamel and Dentin

**DOI:** 10.3389/fphys.2017.00461

**Published:** 2017-07-12

**Authors:** Paula Lancaster, David Brettle, Fiona Carmichael, Val Clerehugh

**Affiliations:** ^1^Restorative Department, School of Dentistry, University of Leeds Leeds, United Kingdom; ^2^Department of Medical Physics and Engineering, St. James's University Hospital Leeds, United Kingdom; ^3^Department of Dental and Maxillofacial Radiology, Leeds Dental Institute, University of Leeds Leeds, United Kingdom

**Keywords:** dental enamel, dental dentin, dental caries, demineralization, thermal imaging, thermal map

## Abstract

The crown of a human tooth has an outer layer of highly-mineralized tissue called enamel, beneath which is dentin, a less-mineralized tissue which forms the bulk of the tooth-crown and root. The composition and structure of enamel and dentin are different, resulting in different thermal properties. This gives an opportunity to characterize enamel and dentin from their thermal properties and to visually present the findings as a thermal map. The thermal properties of demineralized enamel and dentin may also be sufficiently different from sound tissue to be seen on a thermal map, underpinning future thermal assessment of caries. The primary aim of this novel study was to produce a thermal map of a sound, human tooth-slice to visually characterize enamel and dentin. The secondary aim was to map a human tooth-slice with demineralized enamel and dentin to consider future diagnostic potential of thermal maps for caries-detection. Two human slices of teeth, one sound and one demineralized from a natural carious lesion, were cooled on ice, then transferred to a hotplate at 30°C where the rewarming-sequence was captured by an infra-red thermal camera. Calculation of thermal diffusivity and thermal conductivity was undertaken, and two methods of data-processing used customized software to produce thermal maps from the thermal characteristic-time-to-relaxation and heat-exchange. The two types of thermal maps characterized enamel and dentin. In addition, sound and demineralized enamel and dentin were distinguishable within both maps. This supports thermal assessment of caries and requires further investigation on a whole tooth.

## Introduction

The portion of tooth visible within the human mouth, known as the crown, has two main layers of mineralized tissue: enamel and dentin. Tooth enamel is produced by ameloblasts and has the highest mineral content of any tissue within the human body at approximately 96% mineral by weight, compared to dentin which is produced by odontoblasts at approximately 70% mineral by weight (Goldberg et al., 2012; Kunin et al., 2015). Enamel is primarily made of hydroxyapatite, the crystals of which can vary in shape from rods and needles to rhombohedral. They can have a variety of orientations and form enamel prisms. Between the prisms, inter-prismatic crystals, organic material such as proteins, lipids and carbohydrates can be found, as well as water. Enamel mineralization can vary between teeth and within the same tooth. The surface layer of enamel has the greatest level of mineralization, with the layer closest to dentin having the least (Kunin et al., 2015). There are different types of dentin in teeth. The outer layer, mantle dentin, is made of calcospheritic globules with interglobular spaces. Tubules may be found in mantle dentin but is usually void of them. The next layer is circumpulpal dentin, which forms the bulk of the tooth-tissue. It is initially deposited as a cellular layer which matures to predentin and then undergoes mineralization. Tubules are present to house the odontoblast process, with mineralized intertubular dentin between. The circumference of the tubule is made of peritubular dentin, which has a higher mineral content (≈95%) compared to the circumpulpal dentin (≈30%). This peritubular mineral is deposited from the tubular amorphous material. Continuous production of dentin occurs throughout the life of the tooth and, if the tooth is exposed to caries or erosion for example, defensive mechanisms are available, producing either reactionary dentin usually from an odontoblast, or reparative dentin from other cells, e.g., pulp progenitor cells, which is usually void of tubules. Dentin also has an organic matrix (20%) which includes collagens (Types I, III, and V) and non-collagenous proteins, in addition to its water (10%) (Pashley, 1996; Goldberg et al., 2012). Both the mineral layers (enamel and dentin) are heterogeneous and provide protection to the vital soft-tissue - the pulp - centrally. These mineralized tissues are susceptible to dental decay—dental caries—one of the commonest, preventable diseases affecting the human population (Marcenes et al., 2013) where mineral is lost from the tissues and can lead to irreversible cavitation.

Imaging techniques aid detection of dental caries, the simplest being produced from the sensory organ of the eye, which uses the visible light of the electromagnetic spectrum. Light can interact with the mineralized tooth-tissue in a number of ways, such as reflection, scattering, transmission, or absorption. Absorption can produce heat or fluorescence. These interactions can contribute to optical detection methods, such as transillumination, optical coherence tomography and quantitative light-induced fluorescence. There are no health-risks from these methods, which are non-ionizing. Digital imaging fiber optic transillumination (DiFOTI) has limited penetration depth of dental caries but can be improved by using longer wavelengths of near-infrared (780–1550 nm), especially 1,310 nm, due to enamel transparency at these wavelengths. Penetration-depth of optical coherence tomography (OCT) can also be limited but detection of lesions just beyond the dentin-enamel junction have been reported. Mineralized tooth-tissue possesses the ability to auto-fluoresce and quantitative light-induced fluorescence (QLF) uses this property, whereas DIAGNOdent (Kavo) and the LF-pen is thought to use fluorescence from protoporphyrin IX and associated bacterial products, not the mineralized tissue (Hall and Girkin, 2004; Karlsson, 2010; Park et al., 2017).

Over a century ago, the first acceptable dental radiographs for clinical use were reported by Harrison (1896), utilizing X-rays from the electromagnetic spectrum. X-rays have limitations - such as the extent of demineralization needed before caries can be detected (Whaites, 2007) and its location, e.g., occlusal caries (Bader et al., 2002; Bader and Shugars, 2006). Occlusal lesions are positioned in the center of the biting-surface of the tooth and are less easily detected due to the bulk of sound mineralized tissue which surrounds them. This surrounding sound tissue reduces penetration of the X-ray beam, compared to the demineralized lesion which would allow a greater proportion of X-rays to pass onto the image receptor, providing contrast between the lesion and sound tissue. This results in the occlusal lesion being masked by the sound surrounding tissue. X-rays are also ionizing in nature with associated biological risks, e.g., somatic deterministic, somatic stochastic and genetic stochastic effects (Whaites, 2007).

An infra-red thermal camera captures naturally-emitted electromagnetic radiation from the infra-red region. Infra-red radiation has longer wavelengths (700 nm to 1mm) than X-rays (0.01–10 nm), is non-ionizing and totally harmless. The Herschel family were central in discovering infra-red radiation and William Herschel was credited in 1800, and John Herschel produced the first Thermogram in 1840 (Holst, 2000). By the mid-1900s, the military was maximizing the heat-seeking capacity of thermal imaging. Technological advancement in recent years provides accessible and affordable thermal cameras with potential for clinical diagnostic application in medicine. Currently, thermal imaging is not used to detect dental caries but warrants further investigation.

In Kaneko et al. (1999) and Zakian et al. (2010) assessed caries-detection with thermal imaging based on a drop in temperature due to the evaporation of moisture from the porous demineralized tissue compared to sound tissue. Their findings were positive but the thermal properties of the mineralized tissue were not considered. The two mineralized tissues, enamel and dentin, have different compositions and structures (as described earlier) which result in a range of values for thermal diffusivity (enamel ≈2.27–4.69 × 10^−7^m^2^/s, dentin ≈1.83–2.6 × 10^−7^m^2^/s) and thermal conductivity (enamel ≈0.45–0.93 W/mK, dentin ≈ 0.11–0.96 W/mK), as shown in Table 1 (Lin et al., 2010a). These values were obtained from the use of thermocouples, a thermometer, a thermistor, a pulse-laser and infra-red thermography (Lisanti and Zander, 1950; Phillips et al., 1956; Soyenkoff and Okun, 1958; Craig and Peyton, 1961; Braden, 1962; Brown et al., 1989; Panas et al., 2003; Lin et al., 2010b).

**Table 1:** Thermal diffusivity and thermal conductivity values of human enamel and dentin.

	**Enamel**	**Dentin**	**Technique**	**Author**	**Year**
α Thermal Diffusivity (x10^−7^m^2^/s)	4.2	2.6	Thermocouple	Braden	1964
	4.69	1.87-1.83	Thermometer	Brown, Dewey, Jacobs	1970
	2.27	1.92	Pulse Laser and Thermocouple	Panas, Żmuda, Terpilowski, Preiskorn	2003
	4.08	2.01	IR Thermography	Lin, Liu, Kim, Xu, Bai, Lu	2010
κ Thermal Conductivity (W/mK)		0.96	Thermocouple	Lisanti, Zander	1950
		0.11	Thermocouple	Phillips, Johnson, Phillips	1956
		0.45	Thermistor	Soyenkoff, Okun	1958
	0.93	0.58	Thermocouple	Craig, Peyton	1961
	0.92	0.63	Thermocouple	Braden	1964
	0.81	0.48	IR Thermography	Lin, Liu, Kim, Xu, Bai, Lu	2010

A map provides spatial information from co-ordinates on two axes, such as X and Y. The use of a thermal value for each co-ordinate corresponding to a pixel of tooth-tissue could be represented from a grayscale, producing a thermal map specific to that tooth-tissue. This could show the spatial relationship of the thermal properties of the tooth-tissue as an image, rather than a series of numerical values. This may be more clearly understood from the map providing a 2-dimensional relationship of the thermal properties across the whole surface of the tooth-tissue. The thermal properties of enamel and dentin may be sufficiently different to visually distinguish enamel from dentin. Demineralized areas within both tissues may also have different thermal properties due to tissue-changes, such as mineral loss from caries, which may be seen in the thermal map.

The primary aim of this study was to produce a thermal map of a sound, human tooth-slice to visually characterize human enamel and dentin. The secondary aim was to map a human tooth-slice with demineralized enamel and dentin to consider future diagnostic potential of thermal maps in caries-detection.

## Materials and methods

### Materials

Two lower-third molar teeth were ethically sourced from Leeds Dental Institute Research Tissue Bank. Both teeth were cut bucco-lingually with an Accutom-5 (Struers, Copenhagen, Denmark) into 1 mm-thick tooth-slices and then polished with an 800-grit abrasive sheet, with the addition of distilled water as needed. Slice-thickness was measured with a digimatic micrometer (IP65 Quantumike Mitutoyo). Photographs and radiographs of each tooth-slice were taken.

### Method

**Figure 1:**
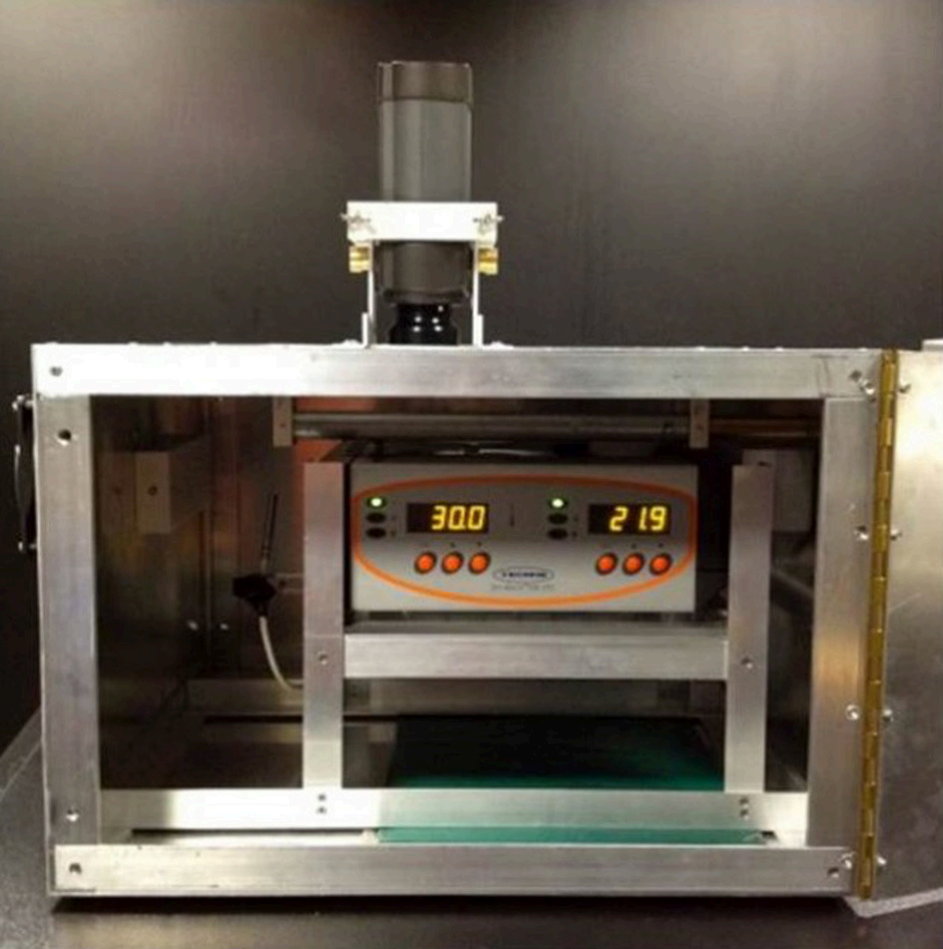
Thermal cube with hotplate and heating-mat (green) in position, with camera secured on the cube by a fixed-mounting normal to the samples.

A purpose-built thermal cube provided a stable thermal environment at 22°C (Figure 1) with macro-regulation from the room air-conditioning and micro-control from a thermal sensor (TD100 Temperature-Controller Auber Instruments) which activated heat-reduction from two thermoelectric cooling units (ActiveCool AC4G TE), or heat-addition from a heating-pad (25 × 35 cm, 2 amp, 15 W, Warmeplatte, BioGreen). A period of 35 min stabilization was allowed prior to commencing data-collection.

A fixed camera-mounting held the FLIR SC305 thermal camera with a x4 lens (resolution of 100 μm) at a focal distance of 8 cm. The parameters used with the camera were: Emissivity 0.96, Reflective Apparent Temperature 27.2°C and Humidity 50%. The recording-rate was 9-frames-per-second and data-collection was with ThermaCAM Researcher Professional 2.10 Software.

An aluminum hotplate (Bibby Techne DB-2TC) was positioned within the cube and stabilized at 30°C. Thermocouples (Omega Type-K SA1XL) were attached to the 0.5 mm thermal pad (thermal conductivity 6 W/mK) of the copper-based specimen-carrier.

Each tooth-slice was paper-dried, placed on the thermal pad and cooled on a block of ice until a temperature of 2°C was recorded on a Fluke 52 II thermometer. The carrier was manually transferred to the aluminum hotplate.

**Figure 2:**
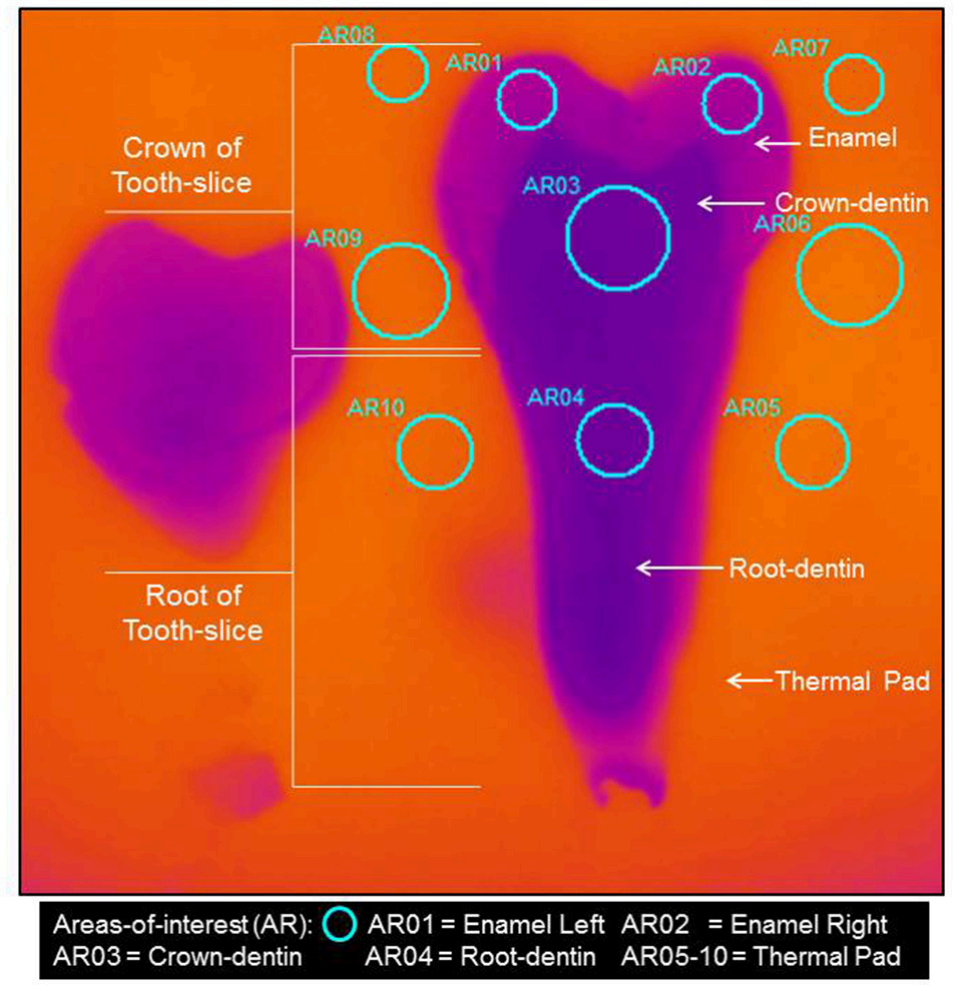
Thermograph of Sample 1–Sound tooth-slice, with circular areas-of-interest shown to enable calculation of the characteristic-time-to-relaxation used in the computation of the thermal diffusivity of the tissues.

Data-capture by the thermal camera commenced prior to transfer of the carrier. Processing of data from selected areas-of-interest circled in blue (Figure 2) occurred initially in a macro-enabled Microsoft Excel File (Microsoft®) to provide time-temperature-curves according to frame-rate. This was followed by selection of the last half of data for curve-fitting to an exponential equation to ascertain the characteristic-time-to-relaxation (τ_*c*_), which required bespoke software for use in Matlab (MathWorks®).

$$\begin{array}{l} \text{f}\left(\text{x}\right)\;=\;\text{C}0*\text{exp}\left(-1*\text{x}/\tau_{c}\right)+\text{C}1 \end{array}$$

The difference in value of characteristic-time-to-relaxation (τ_*c*_) of the thermal pad and the tissue area-of-interest was applied to the formulae below to calculate the thermal diffusivity:

$$\begin{array}{l} \alpha =\frac{4H^{2}}{\pi^{2}\tau_{c}Diff} \end{array}$$

α = Thermal Diffusivity(10^−7^m^2^/Sec)
*H* = Half the thickness of the tissue (m)
τ*_c_Diff* = τ_c_Tissue − τ_c_Thermal Pad (Seconds)

Once the value of thermal diffusivity was known, thermal conductivity could be calculated:

$$\begin{array}{l} \kappa =\;\alpha *\sigma *C_{p} \end{array}$$

κ = Thermal Conductivity (W/mK)
α = Thermal Diffusivity(10^−7^m^2^/Sec)
σ = Density (Kg/m^3^) = 2800 for enamel and 2248 for dentin (Lin et al., 2010b)
*C_p_* = Specific Heat Capacity (J/Kg k) = 710 for enamel and 1066.4 for dentin (Lin et al., 2010b)

The τ_*c*_ and the integral of the curve were calculated for each pixel of the image through the thermal sequence in Matlab (MathWorks®) software, from which two thermal maps were produced.

## Results

### Tooth-slice-thickness

The thickness of the sound tooth-slice measured less than a millimeter in all areas, with the greatest enamel-thickness in the middle at 0.78 mm, the greatest crown-dentin-thickness recorded was on the left at 0.784 mm and the single measurement of the root-dentin was 0.785 mm. The carious tooth-slice was thicker in all areas, with the maximum thickness of enamel at 1.227 mm in the middle, similarly for crown-dentin at 1.223, and the thickest root-dentin was 1.166 mm on the left (see Table 2 for all measurements).

**Table 2:** Dimensions of tooth-slices as recorded with a calibrated digimatic micrometer.

	**Thickness of Teeth-Slices (mm)**								
**Sample**	**Enamel**			**Crown-Dentin**			**Root-Dentin**		
	**Left**	**Middle**	**Right**	**Left**	**Middle**	**Right**	**Left**	**Middle**	**Right**
1	0.776	0.78	0.778	0.784	0.782	0.781		0.785	
2	1.174	1.227	1.161	1.169	1.223	1.166	1.166	1.157	1.164

### Thermal properties

**Figure 3:**
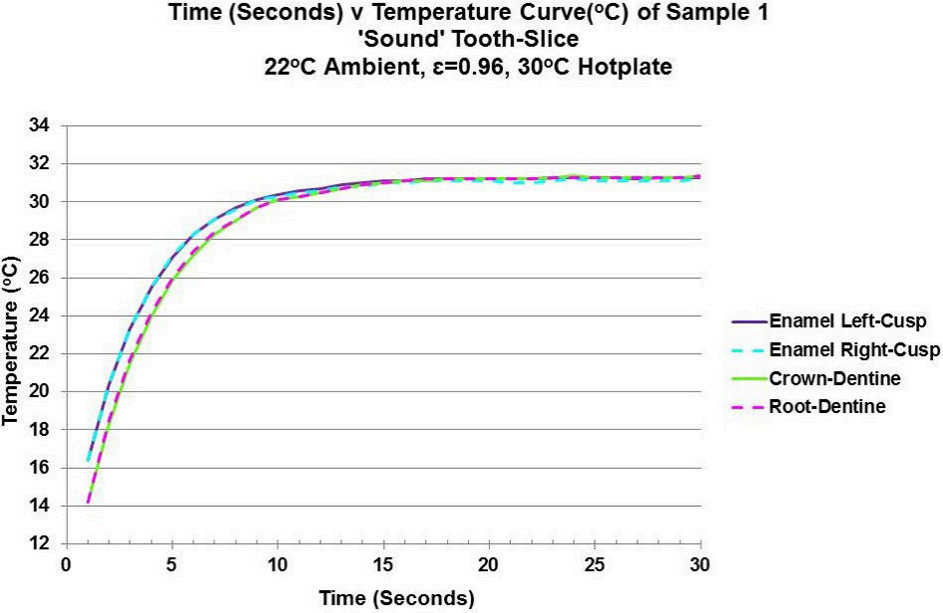
Line Graph of Time (Seconds) v Temperature (°C) showing the data used for curve fitting the exponential equation - f(x) = C0^*^exp(−1^*^x/τ_c_) +C1 - to calculate the characteristic-time-to-relaxation (τ_c_) for enamel and dentin.

The initial rewarming temperatures for the sound tooth-slice in both crown- and root-dentin (green and pink broken-lines) were lower than the two areas of enamel (purple and blue broken-lines). It took circa 30 s to reach thermal equilibrium of all tissues (Figure 3). The rate of rewarming in both regions of enamel are the same and marginally quicker (i.e., a steeper gradient) compared to both crown- and root-dentin which are also the same. The results for the right-hand side enamel and crown-dentin respectively, show differences in characteristic-times-to-relaxation: enamel at 3.655 s (95% CI 3.472, 3.838) compared to crown-dentin at 4.018 s (95% CI 3.855, 4.181), with no overlap in the confidence-intervals for either of these samples. The characteristic-time-to-relaxation (τ_*c*_) is used to calculate the thermal properties of diffusivity and conductivity in conjunction with tissue-thickness. The results indicate from the sound tooth-slice that enamel has a higher value of thermal diffusivity (3.79–4.15 × 10^−7^m^2^/s) and thermal conductivity (0.75–0.83 W/mK) than dentin thermal diffusivity (1.89–2.81 × 10^−7^m^2^/s) and thermal conductivity (0.45–0.67 W/mK) except for one outlier for root dentin with thermal diffusivity of 7.71 × 10^−7^m^2^/s and thermal conductivity of 1.85 W/mK. All values are provided in Table 3. These values fall mainly within the accepted range for thermal diffusivity of enamel at 2.27–4.69 × 10^−7^m^2^/s and dentin at 1.83–2.6 × 10^−7^m^2^/s and thermal conductivity of enamel at 0.45–0.93 W/mK and dentin at 0.11–0.96 W/mK, as previously quoted in Table 1.

**Table 3:** Compilation of sample-thickness as H (m), characteristic-time-to-relaxation (τ_c_ in Seconds), enabling the difference of τ_c_ to be calculated between the thermal pad and area-of-interest, leading to calculation of the thermal diffusivity and, hence, thermal conductivity of enamel and dentin in two slices of tooth - one sound and one carious.

	**H = 0.5 Thickness (m)**	**Tc (Sec)**	**Tc Thermal Pad (Sec)**		**Tc Difference (Sec)**		**Thermal Diffusivity (10^−7^m^2^/Sec) (4H^2^/π^2^Tc Diff) α**		**Density (Kg/m^3^) σ**	**Specific Heat Capacity (J/kg K) cp**	Thermal Conductivity (W/mK) (α^*^σ^*^cp) k	
1-Sound tooth-slice			Left	Right	Left	Right	Left	Right			Left	Right
Lin value enamel							4.08				0.81	
Panas value enamel							2.27				0.45	
Enamel left	0.000388	3.719	3.572		0.147		4.15		2800	710	0.83	
Enamel right	0.000389	3.655		3.493		0.162		3.79	2800	710		0.75
Lin value dentin							2.01				0.48	
Panas value dentin							1.92				0.46	
Crown-dentin	0.000391	4.018	3.69	3.705	0.328	0.313	1.89	1.98	2248	1066.4	0.45	0.47
Root-dentin	0.0003925	3.918	3.696	3.837	0.222	0.081	2.81	7.71	2248	1066.4	0.67	1.85
2-Carious tooth-slice			Left	Right	Left	Right	Left	Right			Left	Right
Lin value enamel							4.08				0.81	
Panas value enamel							2.27				0.45	
Enamel right	0.000587	4.78		4.027		0.753		1.85	2800	710		0.37
Enamel left	0.0005805	4.902	4.027		0.875		1.56		2800	710	0.31	
Enamel caries	0.0006135	5.381	4.027		1.354		1.13		2800	710	0.22	
Lin value dentin							2.01				0.48	
Panas value dentin							1.92				0.46	
Crown-dentin	0.0005845	5.329	3.528		1.801		0.77		2248	1066.4	0.18	
Root-dentin	0.000582	6.001		3.609		2.392		0.57	2248	1066.4		0.14
Dentin caries	0.0006115	5.521	4.027		1.494		1.01		2248	1066.4	0.24	

**Figure 4:**
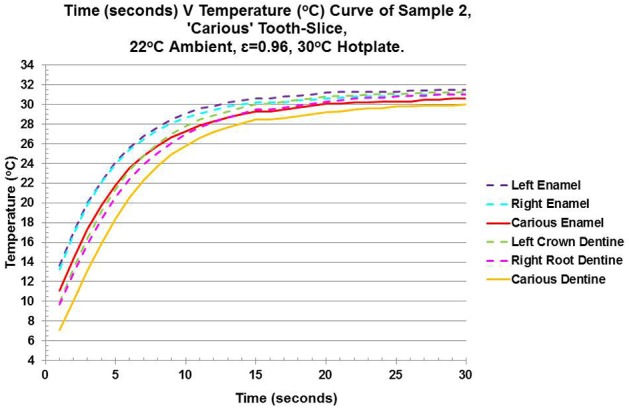
Line Graph of Time (Seconds) v Temperature (°C) showing the data used for curve fitting the exponential equation - f(x) = C0^*^exp(−1^*^x/τ_c_) +C1 - to calculate the characteristic-time-to-relaxation (τ_c_) for enamel, dentin and carious tissue of both enamel and dentin.

Within the carious tooth-slice, the rate of rewarming in the two sound enamel areas-of-interest (purple and blue broken-lines) are similar, whereas the enamel carious lesion is slower (red solid-line) (Figure 4). Carious enamel fails to reach equilibrium in the 30 s time-period. Crown-dentin (green broken-line) warms quicker than root-dentin (pink broken-line). The carious dentin (mustard solid-line) is the slowest of all tissues to rewarm and fails to reach equilibrium within the time-frame. The characteristic-time-to-relaxation for carious enamel is 5.381 s, which is slower than the crown dentin at 5.329 s. This is the only occassion where enamel has a slower characteristic-time-to-relaxation than dentin. All other values give enamel (4.78–4.902 s) a quicker time than dentin (5.521–6.001 s). The enamel has a lower value of thermal diffusivity, ranging from 1.13 × 10^−7^m^2^/s for the carious region to 1.85 × 10^−7^m^2^/s for the right-hand-side sound enamel, than others' findings. Thermal diffusivity shows the crown-dentin (0.77 × 10^−7^m^2^/s) and root-dentin (0.57 × 10^−7^m^2^/s) are reduced, compared to others' findings of 1.83–2.6 × 10^−7^m^2^/s. The carious area of dentin has an increased value of 1.01 × 10^−7^m^2^/s which is comparable to the carious area of the enamel (1.13 × 10^−7^m^2^/s). The thermal conductivity of carious enamel (0.22 W/mK) is similar to carious dentin (0.24 W/mK). All other values of thermal conductivity for enamel and dentin within the carious tooth-slice are lower than others' findings. All values are provided in Table 3.

### Thermal maps

**Figure 5:**
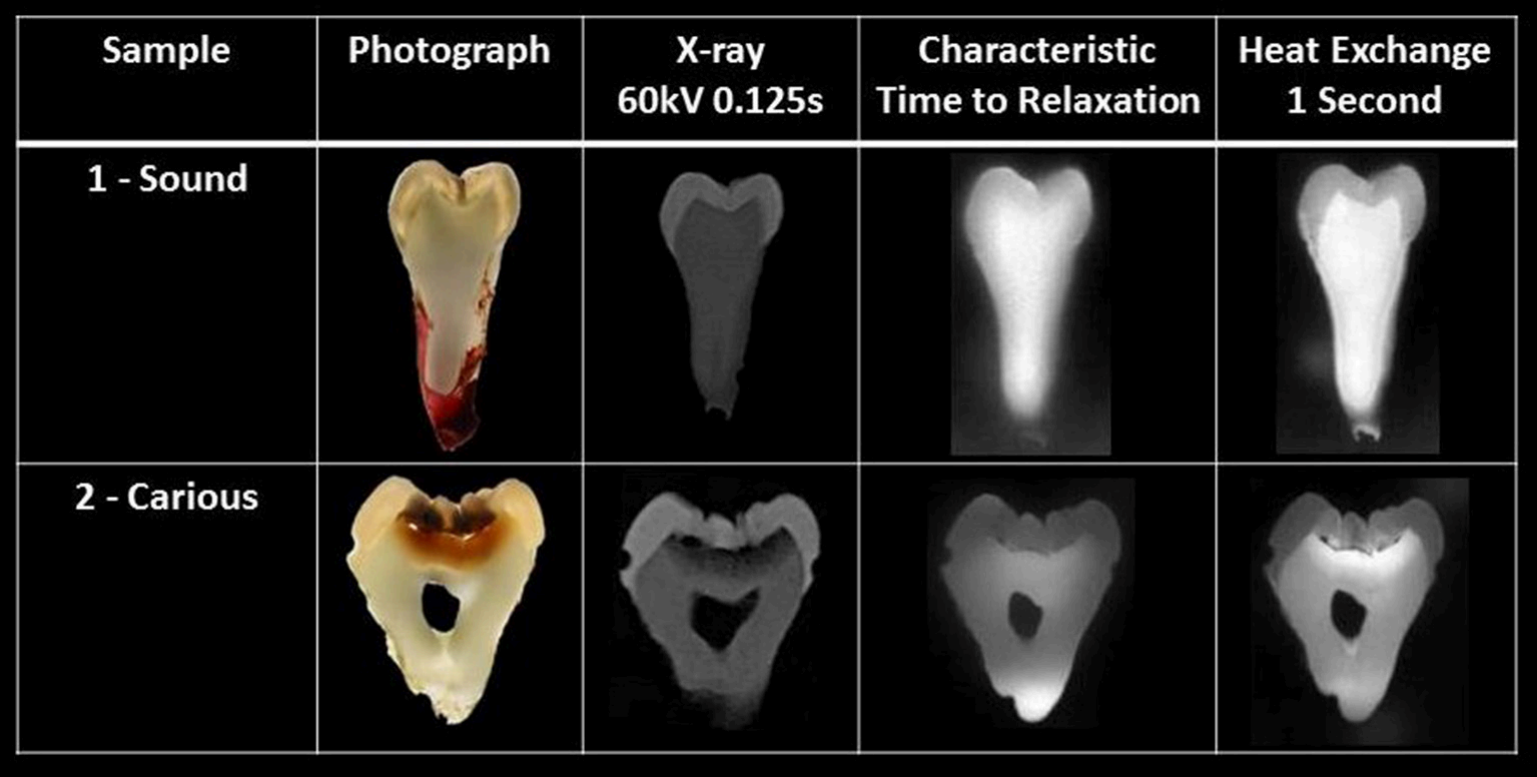
Sample 1- Sound tooth-slice and Sample 2 - Carious tooth-slice. Initially, a photograph is shown, followed by an X-ray, then the characteristic-time-to-relaxation thermal map and, finally, the heat-exchange thermal map depicting enamel, dentin and carious areas of enamel and dentin.

The two thermal maps distinguish the mineralized tissues of enamel, dentin and the carious areas of both tissues using the thermal properties of characteristic-time-to-relaxation and heat-exchange during rewarming (Figure 5).

## Discussion

Infra-red thermal imaging is a technique which is yet to be maximized within the field-of-medicine and its subsidiary specialty - dentistry. Published work for determining the thermal properties of tooth-tissue (Panas et al., 2007; Lin et al., 2010b), was adapted to provide the current methodology. The provision of a stable environment in this study removed environmental temperature-confounders (unlike the previous investigations) and 22°C provided a realistic ambient room-temperature. Tooth-slice thicknesses of 2.2–3.15 mm were used in the previous studies, increasing the three-dimensional heat-transfer compared to the maximum thickness of 1.23 mm within this study. Lin et al. (2010b), applied heat to the occlusal surface of the samples and heat-transfer was recorded along the length of the tooth. Simultaneous heat-application to the irregular occlusal surface would be unlikely, compared to the application of vertical heat to the flat surface of the samples within this study and Panas et al. (2007). The tooth-slices within this study were viewed directly—unlike Lin et al. (2010b), who had applied a black layer of paint. They were also heated directly—unlike Panas et al. (2007), who used a dental cement to attach the samples to the baseplate. Neither of these additional layers was considered in their final calculations. Heat at 30°C was applied in this study to replicate the temperature of the anterior teeth in a living human being (Fanibunda, 1986).

Despite these variations, a single-sample (Lin et al., 2010b), shows comparable results with values reported, as does this study. Multiple samples from different teeth have not previously been reported from this technique, nor have areas of demineralization or caries. All samples are from different donors with inherent anomalies in the tissue-types, as previously described. Investigation of a point location or a single line of a single tissue-sample with any temperature-recording-method, e.g., thermal imaging (Panas et al., 2007; Lin et al., 2010b) or a single thermocouple (Panas et al., 2003) is not ideal. The larger the area-of-interest used for each tissue and the greater the sample-size, the more valid and reliable any inference from the findings. Within this study, two samples, one sound and one carious with a demineralized area, were investigated with multiple areas-of-interest for each tissue. Enamel values of thermal diffusivity and conductivity fall within proximity of known-ranges for the sound tooth-slice but do differ slightly between the two sides examined. Greater variation is seen within the demineralized enamel, where the carious area-of-interest returns the lowest values. This could be explained by the loss of mineral, but caution is needed as the range of values for the sound areas-of-interest differ by a similar proportion within the same sample. These findings appear appropriate to the nature of the tissues being investigated and a more general outcome (as described by Panas et al., 2003) is accepted. That is, following the application of heat and analysis with thermal imaging, a difference between the thermal response of enamel and dentin was detectable, with enamel tending to conduct heat quicker than dentin. The data from this study agrees with that baseline principle and within the two samples presented - sound and demineralized - the thermal properties indicate that enamel conducts heat quicker than dentin within each sample. Two exceptions are seen - one for carious enamel and one for the root-dentin outlier. The carious lesion will have a reduced mineral content - not quantified in this study - and returns a thermal conductivity which lies between the crown-dentin and carious dentin of the same tooth-slice. Comparison between sample-values does not agree with this principle and may be due to the natural variation of the samples from different people, the age of the teeth, the orientation of enamel prisms and dentinal tubules or the carious process. Further work is needed to investigate these relationships.

The purpose of this study was to see if enamel and dentin could be visualized from their individual thermal properties within a map. A thermal map provides a 2-dimensional diagram of the spatial relationship of every thermal value per pixel calculated across the whole tooth-slice. This advances the techniques previously described and adds to the information of an optical image. The thermal maps are produced from the gradient of the rewarming curve - characteristic-time-to-relaxation - and the integral of the curve - heat-exchange. As seen in Figure 5, the two types of thermal map do characterize enamel and dentin.

The characteristic-time-to-relaxation map of sample one, the sound tooth-slice, shows a diffuse boundary between enamel and dentin and is sensitive to the tissue-thickness, as shown from the sloping-sides of the tooth-slice in the root-area. In the second sample, the carious tooth-slice, the slope of the root makes no contact with the thermal pad and is seen clearly as a “white” area at the bottom of the root. This is radiolucent on the X-ray. The thermal map does detect the thin tooth-tissue in the pulp-chamber and produces the outline of the “true” pulp-chamber as seen in the photograph, but this is not seen in the radiographic image where there is insufficient tooth-tissue to attenuate the X-rays. The heat-exchange thermal map shows distinct contrast between enamel and dentin and the carious change within the enamel and dentin is clearly visible, compared to the characteristic-time-to-relaxation thermal map, where there is less contrast of the carious lesion within enamel and diffuse change is seen in dentin. All the advantages of the characteristic-time-to-relaxation thermal map are retained by the heat-exchange thermal map.

The thermal camera and lens used within this study had a spatial resolution of 100 μm producing 5 line-pairs per mm (lp/mm) which was confirmed with a USAF1951 Positive Test Target. OCT has a higher resolution at 5–15 μm (from ≈33 lp/mm), as does DiFOTI at 43 pixels/mm (≈ 21 lp/mm) (Lancaster et al., 2013). The spatial resolution of intra-oral bitewing radiographs has improved from spatial resolutions not dissimilar to those of this thermal camera, up to 20 lp/mm. Unaided human vision is claimed to detect 11 lp/mm on film (Künzel et al., 2003). Phosphor plates can deliver approximately 6–20 lp/mm depending on the age of the plate (Buchanan et al., 2017) and the machine used, e.g., VistaScan or Digora (Li et al., 2008). The spatial resolution defines the ability to distinguish two separate points but this does not necessarily transfer to diagnostic ability for the human operator. The 5 lp/mm available with the thermal camera used produces an acceptable image not dissimilar to that of the digital radiograph from phosphor plates. The lesion shown within the demineralized tooth-slice is large, and the minimum size and level of demineralization detectable with this system is currently unknown and requires additional work with suitable test-objects. Spatial resolution can be limited due to equipment and the infra-red wavelength (700 nm to 1 mm) which will always be less than that of X-rays (0.01 to 10 nm) but may not be a restricting factor for diagnostic ability. This study has viewed slices of teeth *in-vitro*, not a whole tooth, and the findings can underpin future models on whole teeth. Two studies have investigated carious lesions in whole human teeth *in-vitro*- one looking at artificially-created lesions on the smooth labial surface of incisors (Kaneko et al., 1999), and the other viewing naturally-occurring occlusal caries on the surface of molar teeth (Zakian et al., 2010). The theory of a thermal difference between sound tooth-tissue and carious tissue was based on evaporative cooling due to an increase in moisture-content within the micro-pores of the carious lesion. This was found to provide a positive outcome in both studies. Consideration of the thermal properties of the tissues, as seen in this study, were not presented in either of the whole-tooth studies, but their outcomes positively reinforce the need for further work. Cooling by ice to 2°C and rewarming of the samples was observed in this study and there are clinical cooling-methods, such as cryogesic sprays, already in use within dentistry. Water stored in a domestic fridge is approximately 5°C, and the surface-temperature of ice immediately following removal from a domestic freezer is approximately −15°C and, when combined, could provide suitable cooling. This is being investigated for comfort and time-of-application.

The use of thermal imaging to detect approximal caries is unlikely as it cannot penetrate tissues in the way X-rays do. However, detection of early smooth-surface lesions and occlusal lesions would allow preventive measures to be prescribed. X-rays have limitations, as previously mentioned, as do optical detection methods. Thermal imaging may complement our current armamentarium. Detection of active and arrested caries remains uninvestigated with thermal imaging and consideration will be needed for other potential causes of difference in tooth structure and composition, e.g., amelogenesis imperfecta and molar incisor hypomineralization.

## Conclusions

The enamel and dentin of tooth-slices can be characterized *in-vitro* from their thermal properties, as seen in the thermal maps of heat-exchange and characteristic-time-to-relaxation. The heat-exchange map produces better contrast between enamel and dentin than the characteristic-time-to-relaxation map. Within enamel and dentin, demineralized tissue can be detected in both maps, with heat-exchange providing the greatest contrast within both tissues. These thermal maps support further investigation of thermal imaging to complement diagnosis of caries.

## Author contributions

PL, DB, designed the Study, undertook the acquisition, analysis and interpretation of data, wrote the first draft of the manuscript, provided contribution to revision and final approval of the manuscript and are accountable for the work presented. FC, VC, were involved with conception of the design, revision and approval of the manuscript.

### Conflict of interest statement

The authors declare that the research was conducted in the absence of any commercial or financial relationships that could be construed as a potential conflict of interest.

## References

[B1] Bader J. D. Shugars D. A. (2006). The evidence supporting alternative management strategies for early occlusal caries and suspected occlusal dentinal caries. J. Evid. Based Dent. Pract. 6, 91–100. 10.1016/j.jebdp.2005.12.004 17138407

[B2] Bader J. D. Shugars D. A. Bonito A. J. (2002). A systematic review of the performance of methods for identifying carious lesions. J. Public Health Dent. 62, 201–213. 10.1111/j.1752-7325.2002.tb03446.x 12474624

[B3] Braden M. (1962). Heat conduction in teeth and the effect of lining materials. J. Dent. Res. 43, 315–322. 10.1177/00220345640430030201 14159034

[B4] Brown W. S. Dewey W. A. Jacobs H. (1989). Thermal properties of teeth. J. Dent. Res. 49, 752–755. 10.1177/00220345700490040701 5269374

[B5] Buchanan A. Benton B. Carraway A. Looney S. Kalathingal S. (2017). Perception versus reality–findings from a phosphor plate quality assurance study. Oral Surg. Oral Med. Oral Pathol. Oral Radiol. 123, 496–501. 10.1016/j.oooo.2016.12.004 28159585

[B6] Craig R. G. Peyton F. A. (1961). Thermal conductivity of tooth structure, dental cements and amalgam. J. Dent. Res. 40, 411–418. 10.1177/00220345610400030501

[B7] Fanibunda K. B. (1986). The feasibility of temperature measurement as a diagnostic procedure in human teeth. J. Dent. 14, 126–129. 10.1016/0300-5712(86)90077-1 3470335

[B8] Goldberg M. Kulkarni A. B. Young M. Boskey B. (2012). Dentin:structure, composition and mineralization: the role of dentin ecm in dentin formation and mineralization. Front. Biosci. (Elite. Ed). 3, 711–735. 10.2741/e281 PMC3360947 21196346

[B9] Hall A. Girkin J. M. (2004). A review of potential new diagnostic modalities for caries lesions. J. Dent. Res. 83, C89–C94. 10.1177/154405910408301s18 15286130

[B10] Harrison F. (1896). The ‘X’ rays in the practice of dental surgery. Br. Dent. J. 17, 624–628.

[B11] Holst G. C. (2000). Common Sense Approach to Thermal Imaging First. Winter Park, FL: JCD Publishing and SPIE Optical Engineering Press.

[B12] Kaneko K. Matsuyama K. Nakashima S. (1999). Quantification of early carious enamel lesions by using an infrared camera *in-vitro*, in 4th Annual Indiana Conference, ed Stookey G. K. (Indianapolis), 83–100.

[B13] Karlsson L. (2010). Caries detection methods based on changes in optical properties between healthy and carious tissue. Int. J. Dent. 2010:270729. 10.1155/2010/270729 20454579 PMC2864452

[B14] Kunin A. A. Evdokimova A. Y. Moiseeva N. S. (2015). Age-related differences of tooth enamel morphochemistry in health and dental caries. EPMA J. 6:3. 10.1186/s13167-014-0025-8 25685249 PMC4327798

[B15] Künzel A. Scherkowski D. Willers R. Becker J. (2003). Visually detectable resolution of intraoral dental films. Dentomaxillofacial Radiol. 32, 385–389. 10.1259/dmfr/22478778 15070841

[B16] Lancaster P. Carmichael F. Britton J. Craddock H. Brettle D. Clerehugh V. (2013). Surfing the spectrum–what is on the horizon? Br. Dent. J. 215, 401–409. 10.1038/sj.bdj.2013.994 24157760

[B17] Li G. Berkhout W. E. R. Sanderink G. C. H. Martins M. van der Stelt P. F. (2008). Detection of *in-vitro* proximal caries in storage phosphor plate radiographs scanned with different resolutions. Dentomaxillofacial Radiol. 37, 325–329. 10.1259/dmfr/62591340 18757717

[B18] Lin M. Xu F. Lu T. J. Bai B. F. (2010a). A review of heat transfer in human tooth-experimental characterization and mathematical modelling. Dental Mater. 26, 501–513. 10.1016/j.dental.2010.02.009 20303579

[B19] Lin M. Liu Q. D. Kim T. Xu F. Bai B. F. Lu T. J. (2010b). A new method for characterization of thermal properties of human enamel and dentin: influence of microstructure. Infrar. Phys. Technol. 53, 457–463. 10.1016/j.infrared.2010.09.004

[B20] Lisanti V. F. Zander H. A. (1950). Thermal conductivity of dentin. J. Dent. Res. 29, 493–497. 10.1177/00220345500290041201 15436921

[B21] Marcenes W. Kassebaum N. J. Bernabe E. Flaxman A. Naghavi M. Lopez A.. (2013). Global burden of oral conditions in 1990-2010: a systematic analysis. J. Dent. Res. 92, 592–597. 10.1177/0022034513490168 23720570 PMC4484374

[B22] Panas A. J. Preiskorn M. Dabrowski M. Zmuda S. (2007). Validation of hard tooth tissue thermal diffusivity measurements applying an infrared camera. Infrar. Phys. Technol. 49, 302–305. 10.1016/j.infrared.2006.06.021

[B23] Panas A. J. Zmuda S. Terpilowski J. Preiskorn M. (2003). Investigation of the thermal diffusivity of human tooth hard-tissue. Int. J. Thermophys. 24, 837–848. 10.1023/A:1024004803596

[B24] Park K.-J. Haak R. Ziebolz D. Krause F. Schneider H. (2017). OCT assessment of non-cavitated occlusal carious lesions by variation of incidence angle of probe light and refractive index matching. J. Dent. 62, 31–35. 10.1016/j.jdent.2017.05.005 28479506

[B25] Pashley D. H. (1996). Dynamics of the pulpo-dentin complex. Crit. Rev. Oral Biol. Med. 7, 104–133. 10.1177/10454411960070020101 8875027

[B26] Phillips R. W. Johnson R. J. Phillips L. J. (1956). An improved method for measuring the coefficient of thermal conductivity of dental cement. J. Am. Dent. Assoc. 53, 577–583. 10.14219/jada.archive.1956.0217 13366579

[B27] Soyenkoff B. C. Okun J. H. (1958). Thermal conductivity measurements of dental tissues with the aid of thermistors. J. Am. Dent. Assoc. 57, 23–30. 10.14219/jada.archive.1958.0234 13549172

[B28] Whaites E. (2007). Essentials of Dental Radiography and Radiology, 4th *Edn* Edinburgh: Churchill, Livingstone.

[B29] Zakian C. M. Taylor A. M. Ellwood R. P. Pretty I. A. (2010). Occlusal caries detection by using thermal imaging. J. Dent. 38, 788–795. 10.1016/j.jdent.2010.06.010 20599464

